# The Benefits of Collaboration

**DOI:** 10.3390/md21020065

**Published:** 2023-01-19

**Authors:** John W. Blunt, Murray H. G. Munro

**Affiliations:** Department of Chemistry, University of Canterbury, PB 4800, Christchurch 8001, New Zealand

We have been very humbled by the decision of the *Marine Drugs* Editors to honor us with a Special Issue dedicated to our efforts over the past 50 years, as well as their invitation to write a guest editorial for this issue. In 1970 we started working together (collaboration) following our appointments as new academics at the University of Canterbury. The policy of the Department of Chemistry at that time, under Professor Jack Vaughan, was to encourage collaborative, not competitive, research efforts. This policy, when viewed from the lens of 50 years on, seems counter-intuitive when research is very competitive, but at the time, with limited financial resources and students available, it made good sense. Additionally, in our case, it was a good call as we had complementary skill sets and our personalities did not clash. We continued that initial collaborative effort throughout our careers and, to cap it off, we are now writing this editorial collaboratively!

Over the years we have viewed collaboration in a favorable light, and that applies externally as well as between us. Our entry into marine natural products, with a particular emphasis on biological activity/marine invertebrates, came from our collaborations with Professor Ken Rinehart, University of Illinois, in the 1980s, which led to close interactions and collaboration with Dr Michael Boyd’s drug discovery unit, currently the Molecular Targets Program, at the National Cancer Institute in Frederick, Maryland. This inevitably led to collaborations and valuable interactions with Dr Kirk Gustafson, Dr David Newman, and Dr Gordon Cragg in their various roles in the 1990s at the NCI.

On the scientific front, we had a number of successes, with a variety of new skeletons and new bioactive compounds discovered; however, we were also particularly interested in dereplication approaches and endeavored to work on as small a scale as possible. The database, MarinLit, that we developed was instrumental in the development of these approaches. As well as a wide range of bibliographic features, MarinLit also contained NMR features and biogeography data. MarinLit, now maintained by the Royal Society of Chemistry, originally arose from a pile of filing cards transferred to an Apple IIE computer, and was considerably enhanced through collaboration with Professor John Faulkner, who had generated his own marine literature database. The development and maintenance of MarinLit were also instrumental in John and Murray, with three of their former students taking over the annual review of marine natural products published in *Natural Product Reports*, which had been initiated by Professor John Faulkner and maintained by him for 17 years. Sadly, John Faulkner died in 2002, just as our first edition of the review was about to be published. Both John and Murray were then part of the writing team for the review. Murray withdrew from the review after 2016, and John after 2017. The review team is now coordinated by Professor Anthony Carroll.

Collaboration has been an essential part of our academic lives, and we would urge our younger colleagues to think seriously about the advantages of collaborative research with fellow academics rather than following a totally independent pathway.

Finally, we must acknowledge the tremendous contributions made to our efforts from the numerous students, postdoctoral fellows, and other associated workers who have worked with us over the past 50 years. As a result of these efforts we have been honored with both the Paul Scheuer and Norman Farnsworth Awards. We also thank Drs Kirk Gustafson, Shigeki Matsunaga, and David Newman for their very generous comments about us in their introductory editorial for this Special Issue. We have been privileged to have been able to interact with and develop strong friendships with so many other people from all corners of the globe who have also been immersed in the field of marine natural products. The camaraderie and cooperation within this community has been quite remarkable and rewarding to have been a part of.

